# Polymorphisms of the insertion / deletion ACE and M235T AGT genes and hypertension: surprising new findings and meta-analysis of data

**DOI:** 10.1186/1471-2369-6-1

**Published:** 2005-01-11

**Authors:** Adrian Mondry, Marie Loh, Pengbo Liu, Ai- Ling Zhu, Mato Nagel

**Affiliations:** 1 Bioinformatics Institute, 30 Biopolis Street, #07-01 Matrix Building, 138671 Singapore; 2 MolDiag Ag, Albert- Schweitzer- Ring 32, 02943 Weisswasser, Germany

## Abstract

**Background:**

Essential hypertension is a common, polygenic, complex disorder resulting from interaction of several genes with each other and with environmental factors such as obesity, dietary salt intake, and alcohol consumption. Since the underlying genetic pathways remain elusive, currently most studies focus on the genes coding for proteins that regulate blood pressure as their physiological role makes them prime suspects.

The present study examines how polymorphisms of the insertion/deletion (I/D) ACE and M235T AGT genes account for presence and severity of hypertension, and embeds the data in a meta-analysis of relevant studies.

**Methods:**

The I/D polymorphisms of the ACE and M235T polymorphisms of the AGT genes were determined by RFLP (restriction fragment length polymorphism) and restriction analysis in 638 hypertensive patients and 720 normotensive local blood donors in Weisswasser, Germany. Severity of hypertension was estimated by the number of antihypertensive drugs used.

**Results:**

No difference was observed in the allele frequencies and genotype distributions of ACE gene polymorphisms between the two groups, whereas AGT TT homozygotes were more frequent in controls (4.6% vs. 2.7%, P = .08). This became significant (p = 0.035) in women only. AGT TT genotype was associated with a 48% decrease in the risk of having hypertension (odds ratio: 0.52; 95% CI, 0.28 to 0.96), and this risk decreased more significantly in women (odds ratio: 0.28; 95% CI, 0.1 to 0.78). The meta-analysis showed a pooled odds ratio for hypertension of 1.21 (TT vs. MM, 95% CI: 1.11 to 1.32) in Caucasians. No correlation was found between severity of hypertension and a specific genotype.

**Conclusion:**

The ACE I/D polymorphism does not contribute to the presence and severity of essential hypertension, while the AGT M235T TT genotype confers a significantly decreased risk for the development of hypertension in the population studied here. This contrasts to the findings of meta-analyses, whereby the T allele is associated with increased risk for hypertension.

## Background

Essential hypertension is a common, polygenic, complex disorder resulting from interaction of several genes with each other and with environmental factors such as obesity, dietary salt intake, and alcohol consumption. Since the underlying genetic pathways remain elusive[1], currently most studies focus on the genes coding for proteins that regulate blood pressure as their physiological role makes them prime suspects.

The Renin-Angiotensin System (RAS) has a central role in regulating blood pressure and sodium homeaostasis. Genes encoding components of RAS, including angiotensinogen (AGT), angiotensin-converting enzyme (ACE), angiotensinogen II type-1 receptor (AGTR1), and renin, have been extensively investigated as genetic determinants of essential hypertension [2]. Polymorphisms of RAS[3] genes seem also to play a role in the development of diseases that cause secondary hypertension[4,5]. Subjects carrying the ACE D allele have unanimously been shown to have increased ACE serum activity[3,6] while the T235 AGT variant has been associated with elevated angiotensinogen levels [7]. However, so far there are no consistent findings.

In 1992, the M235T AGT TT polymorphism was first reported to be associated with hypertension [8]. This finding has not been confirmed by all investigators[9,10]. Although no relationship between the ACE gene and hypertension was observed in one early linkage study [11] and most recent studies including one meta-analysis [12-14], several studies have suggested a role: hypertensive individuals have a high prevalence of the D allele or DD genotype [3,15,16].

The inconsistent results might be explained in part by the genetic and environmental heterogeneity among different ethnic groups [13]. On the other hand, one recent study [17] reported that the MM, AA, CC, DD/ID genotype combination was associated with a substantially higher prevalence of hypertension in the participants to the Olivetti Heart Study, even though no individual effect of each isolated genotype was detected.

The present study investigates the relationship between variants of the I/D ACE gene and M235T AGT gene, and the presence and severity of essential hypertension in a large homogeneous German population. The effect of a combination of ACE and AGT gene polymorphisms on hypertension was also examined.

## Methods

### Study design

The design of the study followed the guidelines proposed by Cooper et al [18], and the study was carried out in accordance with the Declaration of Helsinki [19].

### Study population

This cross-sectional study comprised a total of 1358 individuals from Weisswasser, a county town of 25,000 in Saxony, Germany. After giving informed consent, 720 normotensive subjects were selected from local blood donors and 638 hypertensive patients from the local renal care center. All hypertensive patients included in the study had been diagnosed as suffering from primary hypertension by the attending consultants on first contact with the clinic. Hypertensives were defined as those who received at least one antihypertensive medication. At the time of blood sampling, 34.2% were diabetic (6 type 1, 212 type 2), and 65.1% were suffering from kidney disease. Of the 37 patients with K/DOQI stage 5 (Kidney failure: GFR, 15 ml/min/1.73 m^2 ^or dialysis), 15 were due to diabetic nephropathy, 5 to chronic pyelonephritis, 1 to chronic glomerulonephritis, 1 light chain deposits, 1 polycystic kidney disease, and 14 of unknown cause (no biopsy obtained). The severity of hypertension was estimated based on the number of antihypertensive medications used, a surrogate marker for the severity of hypertension[8]. Age and gender distribution is described in table 1.

### Genotyping

RFLP (restriction fragment length polymorphism) and restriction analysis were used to determine the frequencies of the I/D polymorphisms of the ACE gene, and homo-/ heterozygoty of the M235T AGT gene[20,21]. ACE I/D polymorphism was studied by PCR based amplification of a 597 bp long gene fragment of the ACE gene, which lacks 287 bp in case of the deletion (D) variant. The primers used were: sense- 5'GATGTGGCCATCACATTCGTCAGAT3', and antisense- 5'CTGGAGACCACTCCCATCCTTTCT3'.

AGT M235T polymorphism was studied by first amplifying a 104 bp long fragment of the AGT gene using the following primer sequences: sense- 5'CCGTTTGTGCAGGGCCTGGCTCTCT3', and antisense: 5'CAGGGTGCTGTCCACACTGGACCCC3'.

The M -> T point mutation at position 235 creates a detection site for the restriction enzyme *Tth 111I*.

### Statistical analysis

Statistical analysis was carried out using SPSS personal computer statistical package (version 11.5, SSPS Inc, Chicago, IL). Demographic characteristics were compared by *t *test for continuous data and Pearson's χ^2 ^test for categorical data. Allele frequencies were calculated with the gene-counting method. χ^2 ^test was used for assessment of the Hardy-Weinberg equilibrium for the distribution of genotypes. Odds ratios were calculated with a 95% confidence interval. A *P *< .05 was considered significant.

### Meta-analysis

A meta-analysis was performed using Review Manager 4.2 (The Cochrane Collaboration) to further examine the association of the AGT M235T gene polymorphisms with essential hypertension in Caucasians. A systematic literature search in PubMed Medline for articles published between April 2002 and June 2004 was carried out using the following MESH-headings: "angiotensinogen/genetics", "hypertension/genetics", "blood pressure/genetics", and "adult". The search was limited to articles published in English and studies on Caucasian human subjects. Only 2 studies were left after strict examination according to the exclusion criteria listed in [22]. A total of 25 studies were finally included: 22 were from the most recent meta-analysis [22], which covered articles from January 1992 to March 2002; 2 were selected from the query described above, and the last one was the present study. Homogeneity among studies was assessed on the basis of χ^2 ^test using P-value < 0.05. The Mantel-Haenszel odds ratios were calculated by applying both fixed effect model and random effect model in case of heterogeneity.

## Results

Demographic data are summarized in Table 1. Hypertensives were older (58.80 ± 13.22 years) than controls (41.24 ± 12.66 years, p < .0001), and more often male (53.2% vs. 44.4%, p = .001).

AGT M/T genotyping was successful in 637 hypertensives and 720 normotensives, and ACE I/D genotype was analyzed in 636 hypertensive and 719 control subjects. The roles of age and gender in the association between hypertension and ACE and AGT gene polymorphisms were examined by comparing the effects of ACE and AGT genotypes for hypertension in men and women, young and elderly subjects respectively. "Young" was defined as "age < 50 years old", and "elderly" was characterized as "age ≥ 50 years old".

### ACE polymorphism

**Figure 1:**
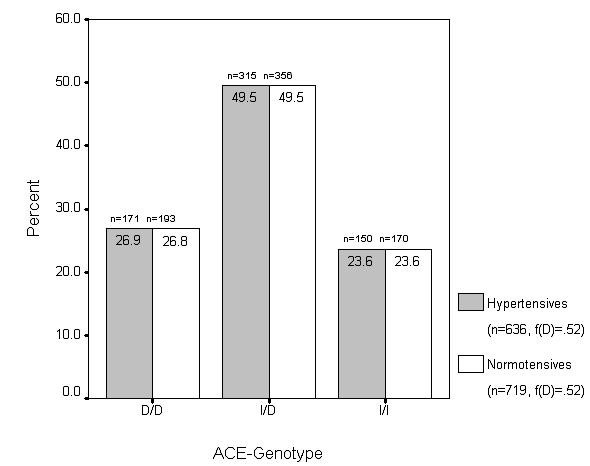
ACE genotypes and allele frequencies, both genders combined. No significant differences. F(D): frequency of D- allele.

No differences were observed in ACE allele and genotype frequency distribution between hypertensives and controls with respect to gender and age, and no deviations from Hardy-Weinberg equilibrium were observed in any of subgroups (P > 0.1). ACE genotypes I/I, I/D and D/D were of almost identical frequency within both groups (P = 1.0, Figure 1). Risk assessment showed that there were no significant risk changes for hypertension in the subjects either with the ACE DD genotype (odds ratio: 1.00, 95% CI: 0.74 to 1.36, P = .98, Table 2) or D allele (odds ratio: D vs. I: 1.00, 95% CI: 0.86 to 1.17, P = .98).

### AGT polymorphism

**Figure 2:**
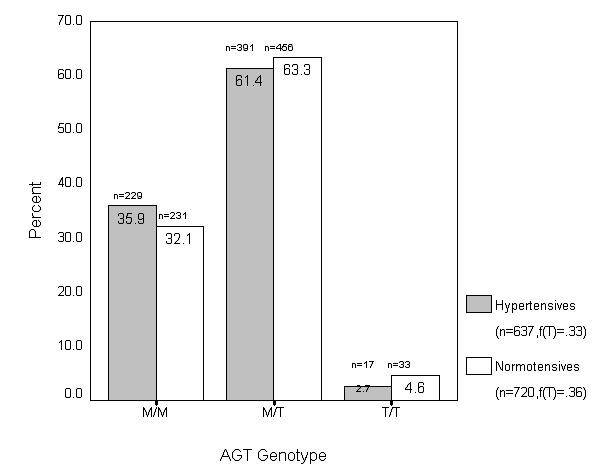
AGT genotypes and allele frequencies, both genders combined. No significant differences. F(T): frequency of T- allele.

**Figure 3:**
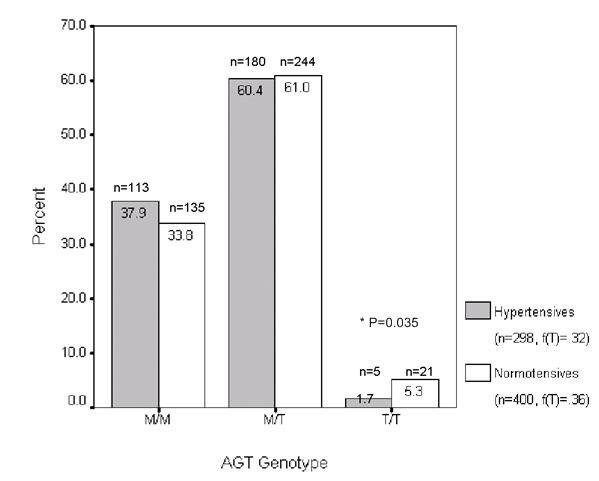
AGT genotypes and allele frequencies in women (significant at p = 0.035). F(T): frequency of T- allele.

AGT T/T homozygotes tended to be more frequent in controls than in hypertensives (4.6% vs. 2.7%, Figure 2). In women, this finding became significant (5.3% vs. 1.7%, Figure 3), but no difference in AGT genotype frequency was found in men. The distribution of the AGT genotypes in all subgroups of the sample population was not in Hardy-Weinberg equilibrium (P < .001). AGT TT genotype was associated with a significant 48% decrease in the risk of being hypertensive (Table 2, odds ratio: 0.52; 95% CI: 0.28 to 0.96; P = .034), and this risk decreased even more to 72% in women (odds ratio: 0.28; 95% CI: 0.1 to 0.78; P = .01). However, no difference was observed in the AGT allele frequency distribution with respect to age and gender (Table 3, P > 0.05), and the effect of the AGT T allele did not reach a significant level in the decrease of hypertension risk (odds ratio: T vs. M: 0.88; 95% CI: 0.75 to 1.03; P = .12).

### Meta-analysis of studies on AGT polymorphisms in Caucasians

**Figure 4:**
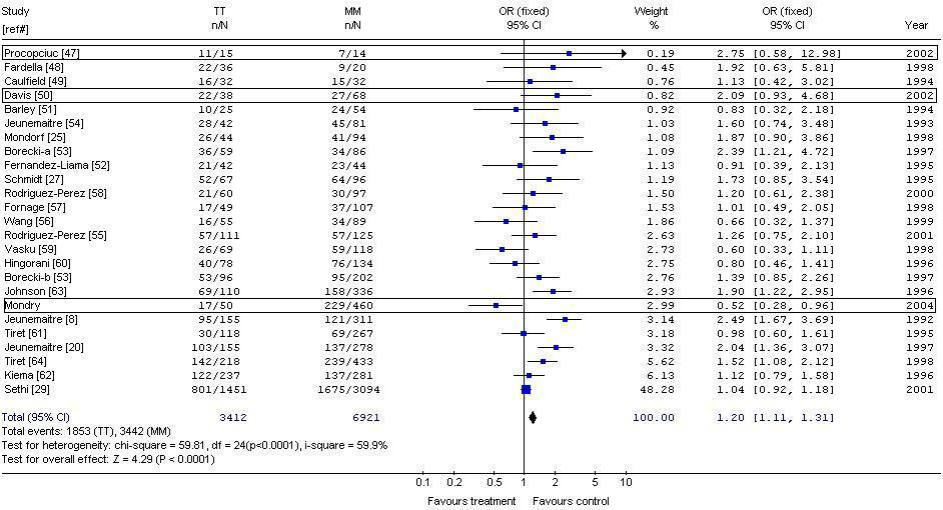
Risk of hypertension associated with the AGT M235T genotypes in Caucasians.

When all studies were pooled, Caucasian individuals with TT genotype had an odds ratio for hypertension of 1.21(95% CI: 1.11 to 1.32) compared with those with MM genotype (Figure 4). The studies included in the meta-analysis are [8,20,23-43]. The pooled odds ratio (odds ratio: TT vs. MM: 1.23; 95% CI: 1.13 to 1.34) increased by 2.5% when the presented study was excluded. Tests for heterogeneity were significant (P < 0.001) in the above two cases, and the odds ratios (TT vs. MM) rose to 1.30 (95% CI: 1.10 to 1.54), and 1.35 (95% CI: 1.15 to 1.59) respectively when applying the random effects model.

### Combination of ACE and AGT polymorphisms

The effect of eight combinations (TT, DD/ID; TT, II/ID; MM, DD/ID; MM, II/ID; DD MM/MT; DD, TT/MT; II, MM/MT; II, TT/MT) on hypertension was examined. No statistically significant association was observed between any combination above and hypertension in all subjects combined. Nonetheless, in women, both genotypes of TT, DD/ID and TT, II/ID were significantly associated with lower prevalence of hypertension (Table 2, 20% vs. 43.3%, odds ratio: 0.33, P = 0.038; 19% vs.43.6%, odds ratio: 0.31, P = 0.026), while MM, DD/ID genotype significantly increased the risk for hypertension (Table 2, 49.2% vs. 40.1%, odds ratio: 1.45, P = 0.028).

No association could be identified between severity of hypertension and a specific ACE or AGT genotype (Table 4).

## Discussion

Although some recent studies [15,16,44,45] suggested a unique sex-specific role of ACE in essential hypertension, no significant association of essential hypertension with the ACE gene I/D polymorphism was observed in this German population of 1,358 for either gender. This finding confirms earlier observations in another German population [31], in other Caucasian populations [11,12,14,46], and in one meta-analysis [13]. The distribution of the ACE genotypes was in Hardy-Weinberg equilibrium in this German population while that was not the case in Pereira et al's study [1]. One possible explanation is the ethnic difference. Pereira et al. [1] showed that there were statistically different ACE I/D polymorphism genotypic frequencies in different ethnic groups.

Surprisingly, the cross-sectional study presented here shows a higher prevalence of the T/T M235T AGT gene in the control group compared to the hypertensive group. AGT TT genotype was associated with a *decrease *in the risk for hypertension (odds ratio-TT vs. MM: 0.52; 95% CI: 0.28 to 0.96) and a more significant association was found in women (odds ratio-TT vs. MM: 0.28; 95% CI: 0.1 to 0.78), compared to men. This is in stark contrast to findings from previous studies, including two German datasets [31,32], and three meta-analyses [10,22,47], which reported that the *AGT *235 T-allele and/or TT genotype significantly *increased *the risk for essential hypertension in Caucasians: odds ratio T vs. M was 1.20 (95% CI: 1.11 to 1.29) [10], odds ratio TT vs. MM was 1.31 [47], and odds ratio TT vs. MM was 1.19 (95% CI: 1.10 to 1.30)[22]. In agreement with the previous meta-analyses, the meta-analysis presented here showed increased odds for hypertension (odds ratio: TT vs. MM: 1.21) in Caucasians conferred by TT, and the odds ratio rose by 2.5% when the present study was excluded. Of the 25 studies included in the present meta-analysis, the present study was the only one in which the AGT T235T genotype decreased odds for hypertension. Nevertheless, the quality of meta-analysis results depends on the quality of the individual studies included, and unusual sample sizes might bias the finding. For example, one single study [43] included in the previously largest meta-analysis [22] was exceptionally large, giving it enormous weight. The highly variable study quality implies that all interpretations must be made with great caution, as was explicitly pointed out by Kunz et al. [10]

Although no significant difference was observed in AGT T allele frequency distribution between hypertensives and controls with respect to age and gender, the frequency of the *AGT *T allele among normotensives was higher than that among hypertensives (0.36 vs. 0.33). This was inconsistent with one previous meta-analysis in Caucasians, which showed that among controls, the mean allele frequency for the *AGT *T allele was 0.41 (95% CI: 0.34 to 0.48), and among cases, increased to 0.45 (95% CI: 0.38 to 0.52) [10]. In the present study, the frequency of the *AGT *T allele among hypertensives (0.33) was outside the lower border of the 95% confidence interval (0.38 to 0.52) reported in [10]. This may reveal the specific genetic background of this particular German population.

The AGT genotype distribution is not in Hardy-Weinberg equilibrium (P < 0.001) while no deviations from Hardy-Weinberg equilibrium are observed on the ACE genotype distribution in the same population. This may be explained by a shift toward a higher frequency of MT individuals (62.4%) instead of TT individuals (3.7%) in this specific population.

The study population presented here contains a large proportion (65%) of patients with renal disease. While selection of participants based on patient records excluded those patients that had symptoms suggesting the diagnosis of secondary hypertension at first contact, the possibility remains that at least a part of the study population suffers from renal rather than essential hypertension. It should be noted, however, that the majority of studies included in the presented meta-analysis does not give specific information regarding renal function of hypertensives, and the largest study [43] is population based and does not name any specific, kidney related exclusion criteria.

Another possible explanation is that the AGT T allele frequency may decrease with age, which was reported from the United Arab Emirates [48]. The prevalence of hypertension increases with advancing age. According to the National Health and Nutrition Examination Survey III (NHANES III) prevalence estimates for the years 1988–1994, American Whites aged 55 to 64 years have a more than threefold higher prevalence of hypertension (42.1%) than those aged 35 to 44 years (11.3%) [49]. Frossard et al detected that AGT T allele frequency decreased with age in the United Arab Emirates[48]. The ACE DD genotype was found associated with human longevity [50]. In the present study, normotensives are significantly younger than hypertensives (41 ± 12 yrs vs. 59 ± 13 yrs). It is conceivable that many of the young individuals are at hypertension risk because of their ACE or AGT genotype, but have not yet shown hypertension at the time of genotyping, and may develop hypertension in their older age. This might lead to some misclassifications and hence reduce the power of this study. On the other hand, two studies of German populations [32,51] reported that the AGT T allele was a risk factor for hypertension in individuals younger than 50 years of age. In the present study, young hypertensives had a higher frequency of the AGT T allele than elderly hypertensives (0.36 vs. 0.32), but the difference didn't reach statistical significance (p = .22). It is possible that the small percentage (24.9%) of the studied hypertensives under 50 years of age biased the finding.

A number of studies [51-54] examined the relationship between RAS genotype and the severity of hypertension, but their results were contradictory. In accordance with two [52,54] of them including one German dataset, the present study fails to find an effect of the AGT or ACE genotype on the severity of hypertension. Nevertheless, in the present study, hypertensives that carried at least one copy of the AGT T allele (TT or MT: n = 397) were less likely to take two or more antihypertensive medications than those with MM genotype (n = 225) (odds ratio -TT or MT vs. MM: 0.797; 95% CI: 0.57 to 1.12; P = .185), and their average number of antihypertensive drugs was lower (2.09 vs. 2.20; P = .276). Despite not reaching statistical significance, this observation was in contrast to Schunkert et al's study [51] on another German population (subjects initially participated in the MONICA Augsburg cohort baseline survey), which found that the carriers of the AGT T allele (n = 418) had a 2.1-fold higher probability of taking two or more antihypertensive drugs than individuals with the MM genotype (n = 216). It is worth pointing out that in the present study, the number of subjects taking antihypertensive medications is much larger than in Schunkert et al's study (622 vs. 143). While the data is far from statistical significance, the trend is in line with those findings that associate rather the M allele with hypertension in the present study.

The effect of a combination of RAAS genes' polymorphisms on blood pressure has been investigated in the participants to the Olivetti Heart Study [17], in which the MM, AA, CC, DD/ID genotype was detected to be associated with a substantially higher prevalence of hypertension in the absence of detectable effects of each individual polymorphism at any single locus. The present study showed very similar findings in women: MM, DD/ID genotype significantly increased the odds for hypertension (odds ratio: 1.45; 95% CI: 1.04 to 2.02), while TT, DD/ID and TT, II/ID were significantly associated with lower prevalence of hypertension (odds ratio: 0.33, 95% CI: 0.11 to 0.99; odds ratio: 0.31, 95% CI: 0.10 to 0.92). The risk of hypertension in the women with TT, DD/ID and TT, II/ID, however, didn't change much compared with those with TT alone (odds ratio: 0.31, 95% CI: 0.12 to 0.83). This suggests that there is only a slight synergistic effect between the AGT and ACE genes.

Although in most surveys the prevalence of hypertension appears to be equal in women and men [55], sex-specific effects of ACE or AGT genes on hypertension have been reported recently [16,43]. For instance, Sethi et al. [43] found the AGT TT associated with an increase in risk for hypertension in women but not in men from the Copenhagen City Heart Study with a population of 9100 subjects, and an association of the ACE DD genotype with increased diastolic blood pressure was detected in men, but not in women from the Framingham Heart Study [16]. In the present study, the AGT TT genotype was negatively correlated to hypertension in women only while no sex-specific effect of the ACE gene was shown. It is possible that the fact is covered by the different gender distributions: in this population, there are more women in normotensives than in hypertensives (55.6% vs. 46.8%, P = .001).

In addition to the factor declared above, the result may be influenced by the study design and the composition of the sample population. The study design itself may influence the results. As mentioned above, this study was a cross-sectional study where subjects were assessed at a single time, and at that time most of the controls were younger than 50 years old. Animal studies have shown that hypertension genes may be activated for only certain periods during the life history of an organism [2]. Hence, some of them might develop hypertension at an older age, resulting from the activated hypertension genes. Longitudinal studies are needed to further examine the relationship between hypertension and genes at different ages.

The population can be described as static population with a mixed Germanic-Slavonic background. Due to the location (a provincial town on the German-Polish border) and political and historical setting (little population fluctuation during the over 140 years of imperial, fascist and socialist rule, no influx due to lack of economic attractivity after reunification), the population may be assumed as homogeneous. Several recent studies [56-61] have reported significant differences in prevalence of hypertension between Germany and Poland, which, however, are assumed to be largely dependent on life-style differences, mostly salt intake. These differences, however, are unlikely to play a role in the study population due to its homogeneity with regards to life style preferences following successful assimilation over many generations. In the case of ACE polymorphism with all allele frequencies greater than 15%, there is no need to examine population stratification [62]. This becomes more urgent for AGT where there is no Hardy- Weinberg equilibrium. As the sample information, however, does not include data on the ethnic background of the probands, additional haplotyping carried out on the samples would not have allowed to rectify for the historical ethnic background (Germanic versus Slavonic).[62,63]

In the meantime, it should be noted that the present study was carried out on an unusually large population (second only to one in 63 studies included in Sethi's meta-analysis[22]). Given the large homogeneous population-based sample, the findings cannot be attributed to simple selection bias. Therefore, the finding that the AGT TT genotype associated with a decreased risk for essential hypertension is likely to be true for this particular German population.

## Conclusions

Despite the limitations mentioned, this cross-sectional study does not support the notion that the ACE I/D polymorphism contributes to the prevalence and severity of essential hypertension. However, the M235T TT genotype of AGT gene was detected to confer a significantly decreased risk for the prevalence of hypertension in women from this particular population. Despite the large sample size, the present study fails to revise the odds ratio in a meta-analysis of a total of 25 studies on the association between the AGT M235T polymorphisms and hypertension in caucasians. This observation may reflect a very specific local inheritance pattern of the AGT genotypes. If this holds true, studies aiming at drug development based on genomic traits must be scrutinized rigorously as therapeutic recommendations may be valid for selected subpopulations only[64].

## Competing interests

The author(s) declare that they have no competing interests.

## Authors' contributions

M.N. designed and initiated the study, analyzed the samples and wrote part of the manuscript. A-L.Z. and M.L. did the statistical analysis. A.M. devised the concept for meta-analysis and wrote the manuscript. L.P. created the figures. M.N. and A.M. should be considered as joint co- authors.

## Pre-publication history

The pre-publication history for this paper can be accessed here:



## Figures and Tables

**Table 1:** Demographic features. M: male. F: female.

	**Hypertensives **(n = 638)	**Normotensives **(n = 720)	P- value
Gender (M/F)	339/298	319/400	0.001
Age (yrs, + SD	58.80 ± 13.22	41.24 ± 12.66	< 0.001
Age < 50 yrs: n (%)	159 (24.9%)	547 (76.0%)	< 0.001
Age > 50 yrs: n (%)	479 (75.1%)	173 (24.0%)	< 0.001

**Table 2:** Hypertension risks estimates for AGT and ACE genotypes by gender.

**Odds ratio **(95%CI)							
MT vs. MM	TT vs. MM	ID vs. II	DD vs. II	MM DD/ID vs. Others	MM II/ID vs. Others	TT DD/ID vs. Others	TT II/ID vs. Others

0.87	0.52	1	1	1.2	1.04	0.56	0.56
(0.69–1.09)	(0.28–0.96)*	(0.77–1.31)	(0.74–1.36)	(0.94–1.53)	(0.81–1.34)	(0.28–1.09)	(0.28–1.12)
0.82	0.83	0.83	0.79	1	1.16	0.85	1.08
(0.59–1.15)	(0.36–1.93)	(0.57–1.21)	(0.52–1.22)	(0.76–1.43)	(0.81–1.66)	(0.34–2.11)	(0.39–3.01)
0.88	0.28	1.26	1.32	1.45	0.97	0.33	0.31
(0.64–1.21)	(0.10–0.78)*	(0.85–1.85)	(0.86–2.03)	(1.04–2.02)*	(0.69–1.37)	(0.11–0.99)*	(0.10–0.92)*

*P < 0.05; CI, confidence interval.

**Table 3:** AGT T allele frequencies by age and gender.

	f(T)		
	
	Hypertensives (n)	Normotensives (n)	P- value
Age < 50 yrs	0.36 (159)	0.36 (547)	NS
Age ≥ 50 yrs	0.32 (479)	0.37 (173)	NS
Male	0.35 (339)	0.37 (319)	NS
Female	0.32 (298)	0.36 (400)	NS
Total	0.33 (638)	0.36 (720)	NS

f(T), frequency of AGT T allele; NS, not significant; yrs, years.

**Table 4:** Prevalence of ACE and AGT genotypes by severity of hypertension.

	**ACE Genotype **n (%)				**AGT Genotype **n (%)			
	
	II	ID	DD	P- value	MM	MT	TT	P- value
Hypertensive, one drug	56 (22)	120 (48)	74 (30)	0.458	83 (33)	163 (65)	5 (2)	0.225
Hypertensive, > two drugs	89 (24)	189 (51)	93 (25)	0.458	142 (38)	217 (59)	12 (3)	0.225
